# The TERB1 MYB domain suppresses telomere erosion in meiotic prophase I

**DOI:** 10.1016/j.celrep.2021.110289

**Published:** 2022-01-25

**Authors:** Kexin Zhang, Agata Tarczykowska, Deepesh Kumar Gupta, Devon F. Pendlebury, Cassandra Zuckerman, Jayakrishnan Nandakumar, Hiroki Shibuya

**Affiliations:** 1 Department of Chemistry and Molecular Biology, University of Gothenburg, Gothenburg SE-41390, Sweden; 2 Department of Molecular, Cellular, and Development Biology, University of Michigan, Ann Arbor, MI 48109, USA; 3 Program in Chemical Biology, University of Michigan, Ann Arbor, MI 48109, USA; 4 Present address: Pharmaceutical Sciences, University of California, Irvine, Irvine, CA, USA; 5 Lead contact

## Abstract

The meiosis-specific telomere-binding protein TERB1 anchors telomeres to the nuclear envelope and drives chromosome movements for the pairing of homologous chromosomes. TERB1 has an MYB-like DNA-binding (MYB) domain, which is a hallmark of telomeric DNA-binding proteins. Here, we demonstrate that the TERB1 MYB domain has lost its canonical DNA-binding activity. The analysis of *Terb1* point mutant mice expressing TERB1 lacking its MYB domain showed that the MYB domain is dispensable for telomere localization of TERB1 and the downstream TERB2-MAJIN complex, the promotion of homologous pairing, and even fertility. Instead, the TERB1 MYB domain regulates the enrichment of cohesin and promotes the remodeling of axial elements in the early-to-late pachytene transition, which suppresses telomere erosion. Considering its conservation across metazoan phyla, the TERB1 MYB domain is likely to be important for the maintenance of telomeric DNA and thus for genomic integrity by suppressing meiotic telomere erosion over long evolutionary timescales.

## In brief

The meiosis-specific telomere protein TERB1 anchors telomeres to the nuclear envelope and drives chromosome movement. Zhang et al. show that the MYB domain of TERB1 facilitates the localization of chromosomal axis proteins at telomeres and protects telomeres from meiotic telomere erosion, which likely ensures genomic integrity over long evolutionary timescales.

## Graphical Abstract

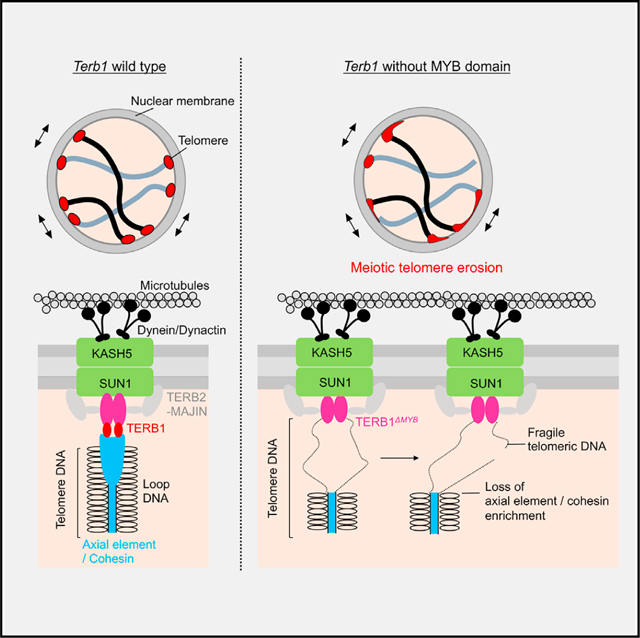

## INTRODUCTION

Telomeres, which are found at the ends of linear eukaryotic chromosomes, are composed of G-rich repeat DNA sequences— (TTAGGG)_n_ in vertebrates. Proteins that bind to telomeric double-strand DNA (dsDNA) typically have a single MYB-like DNA-binding (MYB) domain at their C terminus that directly recognizes the telomeric dsDNA in a sequence-specific manner, such as Taz1 in fission yeast, RTBP1 in rice, and TRF1/2 in mammals (Broccoli et al., 1997; Spink et al., 2000; Yu et al., 2000). There are several exceptions, such as Rap1 in budding yeast and DTN-1/2 (also known as TEBP-1/2) in nematodes, which have two and three tandem MYB domains, respectively (Bilaud et al., 1996; Konig et al., 1996; Krauskopf and Blackburn, 1996; Yamamoto et al., 2021). The recognition of telomeric dsDNA via these MYB domain proteins leads to the assemblies of downstream telomere-associating proteins, forming the so-called shelterin complex, which ensures the fundamental telomeric functions, such as chromosome end protection and telomere length maintenance (de Lange, 2010).

In addition to these housekeeping functions, telomeres have an evolutionarily conserved role during meiosis. In meiotic prophase I, homologous chromosomes are paired and recombined, which is ensured by chromosome movement along the nuclear envelope (NE) (Koszul and Kleckner, 2009). To drive the movements, meiotic telomeres attach to the NE, interact with the transmembrane linker of nucleoskeleton and cytoskeleton complex (the SUN1-KASH5 complex in mammals), and become linked to the cytoskeletal motor proteins in a transmembrane manner (Ding et al., 2007; Hiraoka and Dernburg, 2009; Horn et al., 2013; Morimoto et al., 2012). A conserved germ-cell-specific telomere-binding protein, TERB1, is central in this meiosis-specific role in metazoans (da Cruz et al., 2020; Shibuya et al., 2014). TERB1 binds to the shelterin protein TRF1 through its TRF1-binding (TRFB) domain, which localizes TERB1 to telomeres, where it recruits the downstream transmembrane components TERB2-MAJIN and SUN1-KASH5 for telomere attachment and movement, respectively. Accordingly, deletion of any of these genes (i.e., *Terb1*, *Terb2*, *Majin*, *Sun1*, or *Kash5*) results in complete infertility in mice in both sexes (Ding et al., 2007; Horn et al., 2013; Shibuya et al., 2014, 2015). Recent studies have identified pathological mutations in *TERB1*, *TERB2*, and *MAJIN* in human non-obstructive azoospermia patients, corroborating these genes’ critical roles in human reproduction (Alhathal et al., 2020; Riera-Escamilla et al., 2020; Salas-Huetos et al., 2021).

Notably, TERB1 has a single MYB domain at its C terminus, which is similar to the canonical telomeric dsDNA-binding proteins, thus implying their monophyletic evolutionary origin (Shibuya and Watanabe, 2014). However, the role of the TERB1 MYB domain has been unclear. The TRFB domain of TERB1, which is adjacent to the MYB domain, is necessary and sufficient for localizing TERB1 to telomeres, suggesting that, unlike TRF1 and TRF2, the MYB domain of TERB1 seems to be dispensable for telomere localization (Pendlebury et al., 2017; Shibuya et al., 2014). The analysis of *Trf1* conditional knockout mice showed that the telomere localization of TERB1 depends on TRF1 (Zhang et al., 2017), further reinforcing the notion that the protein-protein interaction between TRF1 and the TRFB domain is the major pathway for recruiting TERB1 to the meiotic telomeres, leaving the function of the TERB1 MYB domain unknown.

In this study, by screening founder mice with random point mutations introduced by CRISPR-Cas9, we isolated a mutant allele with an in-frame premature stop codon in the *Terb1* gene. This gene expresses a TERB1 protein lacking the MYB domain but retaining all other functional elements of TERB1. Through our mouse studies, we have unambiguously clarified the role of the MYB domain in TERB1 as the guardian of telomeric DNA from aberrant erosion during meiotic chromosome movements.

## RESULTS

### The TERB1 MYB domain has lost its DNA-binding activity

**Figure 1.**
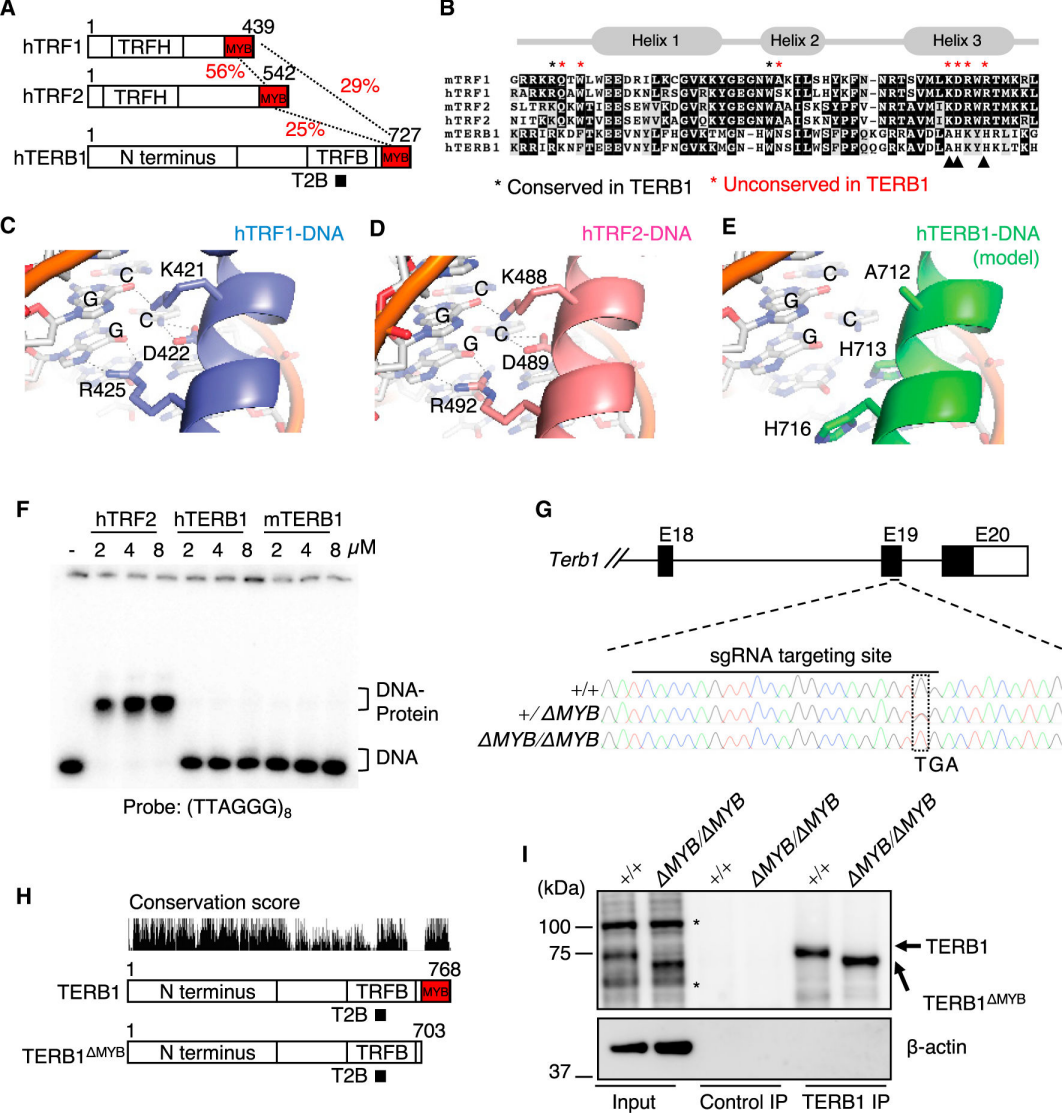
Generation of *Terb1^ΔMYB^* mice (A) Schematic of human TRF1, TRF2, and TERB1 highlighting the TRF homology (TRFH) domain, MYB domain, TRF1-binding (TRFB) domain, TERB2-binding (T2B) domain, and TERB1-specific N-terminal extension. The percentage of amino acid identity between the MYB domains is shown. (B) Sequence alignment of the MYB domains from mouse and human TRF1, TRF2, and TERB1. Amino acids that directly contact telomeric dsDNA identified in human TRF1 are highlighted by asterisks (residues shown in red are not conserved in TERB1). Residues critical for sequence-specific binding to the telomeric major groove in the third recognition helix in TRF1/2 are highlighted by arrowheads. (C–E) Views of the MYB-DNA interfaces of hTRF1 (C), hTRF2 (D), and hTERB1 homology model (E) with side chains involved in base-specific contacts in the major groove shown as sticks. Dashed lines indicate H-bonds between protein and DNA. (F) EMSA with isolated MYB domains from hTRF2, hTERB1, and mTERB1. (G) Schematic of the *Terb1^ΔMYB^* alleles. Exons are shown as rectangles, and the protein coding regions are marked by black rectangles. DNA sequencing results are shown. (H) Schematic of the TERB1^ΔMYB^ protein with the conservation score. (I) Immunoprecipitates with the control or TERB1 antibodies from WT and *Terb1^ΔMYB/ΔMYB^* testis extracts. Asterisks are non-specific bands.

The sequence alignment of the MYB domain of human TERB1 with the MYB domains of TRF1 and TRF2 confirmed the significant sequence similarities (Figure 1A: 25% and 29% identities between the TRF2 and TERB1 MYB domains and between the TRF1 and TERB1 MYB domains, respectively). The structural alignment of their MYB domains corroborated their excellent overall homology (Figure S1A), but the amino acids directly contacting the telomeric dsDNA, as characterized in human TRF1/2 (Court et al., 2005), are poorly conserved in both mouse and human TERB1 (asterisks in Figure 1B). The DNA-binding interfaces in the third recognition helix show that the lysine, aspartate, and arginine residues that are critical for sequence-specific binding to the telomeric major groove in TRF1/2 are substituted with alanine, histidine, and histidine residues, respectively, in TERB1 (Figures 1C–1E). Because the single substitution of arginine 425 to valine in human TRF1 is sufficient to abrogate the dsDNA-binding activity (Fairall et al., 2001), we hypothesized that the dsDNA-binding activity was lost in the TERB1 MYB domain. To directly test this, we conducted an electrophoretic mobility shift assay (EMSA) using telomeric dsDNA probes mixed with *in vitro* purified MYB domains. The EMSA showed that the isolated MYB domain of human TRF2 (hTRF2-MYB) had dsDNA-binding activity as assessed by the gradual increase of ladder-like upshifted bands with increased protein concentration, consistent with gradual saturation of the probe DNA with the hTRF2-MYB proteins (Figure S1B). At higher concentrations, the protein-DNA complex collapses into a single band, which is representative of telomeric DNA being fully saturated with the protein (Figure S1B). However, the human and mouse TERB1 MYB domains did not bind to telomeric repeat dsDNA (Figure 1F) or non-telomeric DNA (Figure S1C), even at protein concentrations that were sufficient to fully saturate the telomeric DNA probe with hTRF2-MYB. We then asked whether restoring the three critical DNA-binding residues at the recognition helix in TERB1 MYB would restore DNA binding. We generated three single substitutions and a triple mutant of TERB1 and performed EMSA but did not see any evidence of DNA binding (Figures S1D and S1E). These results suggest that the changes to the TERB1 MYB domain go beyond the substitution of amino acid side chains involved in DNA binding and likely lead to new, perhaps protein-protein binding, functions.

### Generation of Terb1*^△MYB/△MYB^* mice

To study the function of the TERB1 MYB domain, we used CRISPR-Cas9 to introduce random mutations in the mouse *Terb1* gene in exon 19, which is upstream of the MYB-domain coding region. After screening of the founder mice, we obtained an allele where the codon for glycine 704 was substituted with a premature stop codon (Figure 1G). In principle, this in-frame mutant allele codes for a truncated TERB1 protein (1–703 amino acids) containing all of the functional domains so far identified, such as the N-terminal domain (which binds to SUN1), the TRFB domain, and the TERB2-binding domain, but lacking the C-terminal MYB domain (Figure 1H). We refer to this allele and the corresponding protein product as *Terb1^ΔMYB^* and TERB1^ΔMYB^, respectively.

The introduction of a premature stop codon by chance can activate nonsense-mediated mRNA decay, leading to the abrogation of protein expression (Kurosaki and Maquat, 2016). To study the protein expression from our *Terb1^ΔMYB^* allele, we performed TERB1 immunoprecipitation (IP) from testis extracts from homozygous mice (*Terb1^ΔMYB/ΔMYB^*) and wild-type (WT) controls. Immunostaining with the TERB1 antibody yielded a band close to the 75-kDa marker in WT extracts (close to the theoretical molecular weight of TERB1 of 87 kDa), whereas a slightly faster migrating band was detected in *Terb1^ΔMYB/ΔMYB^* extracts (Figure 1I). The band with faster migration is likely to be the TERB1^ΔMYB^ protein (with a theoretical molecular weight of 79 kDa). Indeed, these bands were specifically enriched after IP with TERB1 antibody (Figure 1I), indicating that the *Terb1^ΔMYB^* allele escapes nonsense-mediated mRNA decay and expresses the TERB1^ΔMYB^ protein. The expression level of TERB1^ΔMYB^ was almost equal to that of WT protein, as seen by the comparable intensities of these bands, thus making these mice suitable for the domain-specific functional analysis of TERB1.

### Telomere attachment to the NE occurs in Terb1*^△MYB/△MYB^* mice

**Figure 2.**
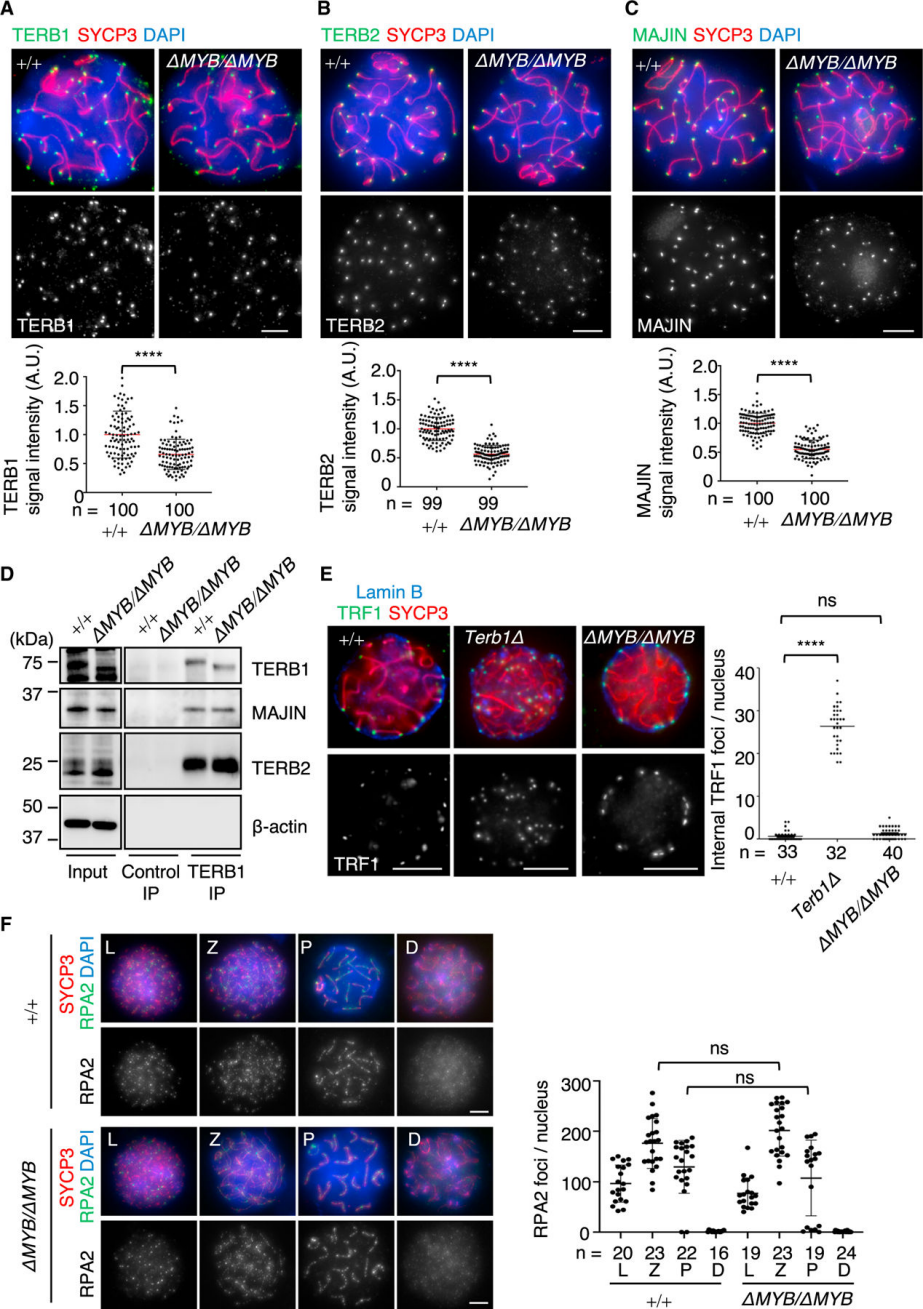
Telomere attachment to the NE is intact in *Terb1^ΔMYB^* mice (A–C) TERB1 (A), TERB2 (B), and MAJIN (C) were stained with SYCP3 in WT and *Terb1^ΔMYB/ΔMYB^* spermatocytes. The graph shows the mean signal intensity with standard deviations normalized to the mean value of WT spermatocytes. n shows the number of telomeres pooled from five pachytene cells. Scale bars, 5 μm. All analyses used two-tailed t tests. ****p < 0.0001. (D) Immunoprecipitates with the control or TERB1 antibodies from WT and *Terb1^ΔMYB/ΔMYB^* testis extracts. (E) Immunostaining of WT, *Terb1Δ* (null), and *Terb1^ΔMYB/ΔMYB^* spermatocytes. Equatorial sections are shown. The graph shows the mean internal TRF1 foci number with standard deviations. n shows the number of spermatocytes (pachytene spermatocytes for WT and *Terb1^ΔMYB/ΔMYB^* and zygotene spermatocytes for *Terb1Δ*). Scale bars, 5 μm. ns, not significant; ****p < 0.0001 by one-way ANOVA. (F) Immunostaining of WT and *Terb1^ΔMYB/ΔMYB^* spermatocytes. The graph shows the number of RPA2 foci associated with the chromosome axes. The mean values with SD are shown, and n shows the number of cells pooled from two mice for each genotype. D, diplotene; L, leptotene; P, pachytene; and Z, zygotene. Scale bars, 5 μm. All analyses used two-tailed t tests.

Immunostaining of TERB1 protein in spermatocyte spreads showed distinct punctate foci at the ends of SYCP3-stained chromosome axes in both WT and *Terb1^ΔMYB/ΔMYB^* mice (Figure 2A). The average number of foci was around 40 in both WT and *Terb1^ΔMYB/ΔMYB^* mice (Figure S2A), which corresponded to the number of telomeres and suggested that TERB1^ΔMYB^ localizes to telomeres in a similar manner as the WT protein. The downstream telomeric proteins TERB2 and MAJIN were also localized at the ends of the chromosome axes in *Terb1^ΔMYB/ΔMYB^* spermatocytes (Figures 2B, 2C, and S2A). This is reasonable because TERB2-MAJIN binds to the TERB2-binding (T2B) domain of TERB1, which resides next to the MYB domain and is intact in the TERB1^ΔMYB^ protein (Figure 1H; Dunce et al., 2018; Zhang et al., 2017). TERB1 IP and western blotting confirmed that both TERB2 and MAJIN were similarly precipitated with TERB1^ΔMYB^ and with WT protein (Figure 2D), proving that the TERB1-TERB2-MAJIN ternary complex formation is not affected by the absence of the MYB domain. It is noteworthy that the signal intensities of TERB1/TERB2/MAJIN foci were lower in *Terb1^ΔMYB/ΔMYB^* spermatocytes compared with WT, suggesting that the MYB domain contributes to the stabilization of TERB1 as well as the downstream TERB2/MAJIN complex at meiotic telomeres, even though the contribution is minor (Figures 2A–2C).

The residual localization of the TERB1-TERB2-MAJIN ternary complex in *Terb1^ΔMYB/ΔMYB^* spermatocytes prompted us to investigate the telomere attachment states in this mutant. The triple staining of TRF1, SYCP3, and the NE marker Lamin B confirmed that most of the telomeres attached to the NE in *Terb1^ΔMYB/ΔMYB^* spermatocytes similarly to WT spermatocytes (Figure 2E). This is unlike *Terb1*-null mice, where a significant number of telomeres detached from the NE (Figure 2E; Shibuya et al., 2014). Furthermore, there was no increase in apoptotic cells in *Terb1^ΔMYB/ΔMYB^* seminiferous tubules (Figure S2B), and the progression of meiotic prophase I seen by the staining of SYCE3, a marker of homologous synapsis, was normal in *Terb1^ΔMYB/ΔMYB^* spermatocytes (Figure S2C). Together, these results lead to the conclusion that the TERB1 MYB domain is dispensable for the establishment of telomere-NE attachment and homolog pairing and synapsis.

### Progression of meiotic recombination is normal in Terb1*^△MYB/△MYB^* mice

Telomere attachment to the NE facilitates the chromosome movements that are required for the recombination of homologous chromosomes. Therefore, we analyzed whether the progression of meiotic recombination is affected in *Terb1^ΔMYB/ΔMYB^* spermatocytes by staining for RPA2, which binds to the resected single-strand DNA after the induction of programmed double-strand breaks (DSBs) at the sites of meiotic recombination. In WT spermatocytes, RPA2 foci began to appear along the chromosome axis in the leptotene stage, the number of foci peaked in the zygotene stage, and the foci gradually disappeared toward the pachytene to diplotene stages in accordance with the gradual repair of the DSBs (Figure 2F). In *Terb1^ΔMYB/ΔMYB^* spermatocytes, the spatiotemporal distribution of RPA2 foci was indistinguishable from WT spermatocytes, suggesting that the induction of meiotic DSBs and their repair by homologous recombination were unaffected (Figure 2F). Consistent with this, the staining of MLH1, which marks the destined crossover sites in late-pachytene spermatocytes, confirmed the presence of a comparable number of foci in *Terb1^ΔMYB/ΔMYB^* spermatocytes (Figure S2D). These results suggest that meiotic recombination is not affected in *Terb1^ΔMYB/ΔMYB^* spermatocytes.

### The TERB1 MYB domain suppresses telomere erosion

**Figure 3.**
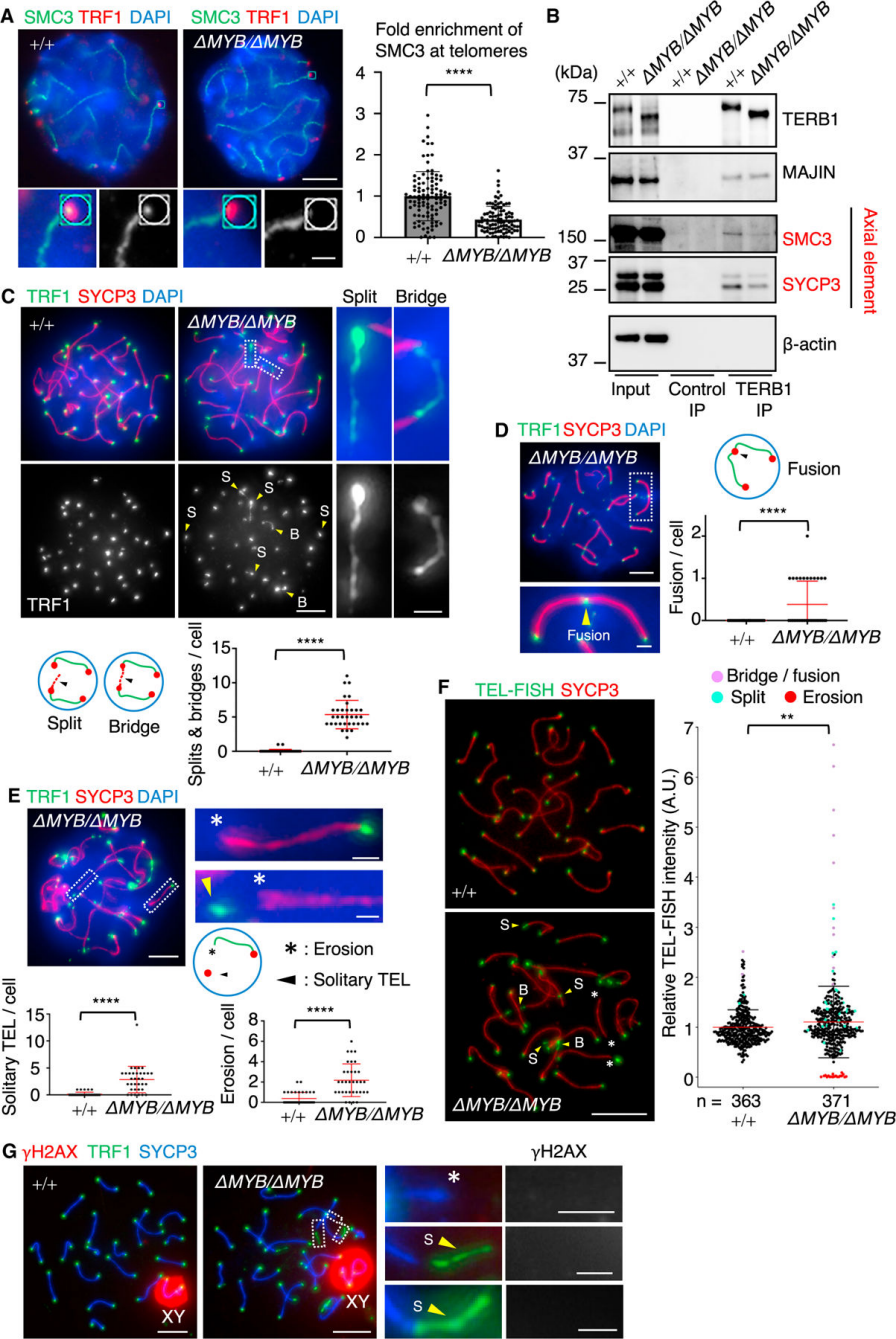
Telomere structural defects in *Terb1^ΔMYB/ΔMYB^* mice (A) Immunostaining of WT and *Terb1^ΔMYB/ΔMYB^* pachytene spermatocytes. The graph shows the mean signal intensity of SMC3 at the telomeres normalized to the mean value of WT. A total of 100 telomeres pooled from 10 pachytene cells were used for the quantification for each genotype. Scale bar, 5 μm (1 μm in the magnified panel). All analyses used two-tailed t tests. ****p < 0.0001. (B) Immunoprecipitates with the control or TERB1 antibodies from WT and *Terb1^ΔMYB/ΔMYB^* testis extracts. (C–E) Immunostaining of WT and *Terb1^ΔMYB/ΔMYB^* pachytene spermatocytes. The mean numbers of splits (S) and bridges (B) per cell and the mean numbers of fusion, erosion, and solitary telomeres per cell were quantified in (C), (D), and (E), respectively. n = 34 cells, and error bars are the standard deviation. Scale bar, 5 μm (1 μm in the magnified panel). All analyses used two-tailed t tests. ****p < 0.0001. (F) Fluorescence *in situ* hybridization of WT and *Terb1^ΔMYB/ΔMYB^* pachytene spermatocytes, stained with SYCP3 antibody, hybridized with telomeric probe. S, B, and erosion (asterisk) are indicated. Scale bar, 5 μm. The graph shows the quantification of individual telomeric fluorescence *in situ* hybridization (FISH) signal intensities. The average values are normalized to that of WT. n shows the analyzed number of telomeres from 10 (WT) to 11 (*Terb1^ΔMYB/ΔMYB^*) nuclei. The median with variability and probability density is shown. The distributions were significantly different between WT and *Terb1^ΔMYB/ΔMYB^* (two-tailed Mann-Whitney test; **p < 0.01). (G) Immunostaining of WT and *Terb1^ΔMYB/ΔMYB^* pachytene spermatocytes. Telomeres with S and erosion (asterisk) are magnified. Scale bars, 5 μm (1 μm in the magnified panel).

During meiosis, the cohesin complex forms the chromosome axis and TERB1 plays a role in the enrichment of the cohesin axial core at telomeres in order to maintain the structural rigidity of telomeres (Shibuya et al., 2014). We examined whether this pathway is intact in *Terb1^ΔMYB/ΔMYB^* spermatocytes by staining for SMC3, which is a common subunit in all meiotic cohesin subcomplexes (Ishiguro et al., 2011). We found that the localization of SMC3 in the chromosomal arm region was intact, but the amount of SMC3 at the telomeres was significantly reduced in *Terb1^ΔMYB/ΔMYB^* spermatocytes (Figure 3A). coIP with the TERB1 protein from WT and *Terb1^ΔMYB/ΔMYB^* spermatocytes showed reduced binding of SMC3 to TERB1^ΔMYB^ compared with WT protein, suggesting that the MYB domain is required for the stable interaction with the cohesin complex (Figure 3B).

The loss of meiotic cohesin from telomeres is known to induce structural abnormalities in telomeres (Adelfalk et al., 2009). Indeed, there were significant structural defects as seen by staining for TRF1 in *Terb1^ΔMYB/ΔMYB^* spermatocytes, and the TRF1 signal stretched from the end of the chromosome axis (referred to as a split) and was occasionally connected to the telomeres of non-homologous chromosomes (referred to as a bridge; Figure 3C). The average number of splits and bridges was 5.4 per spermatocytes, and all spermatocytes observed had at least one split or bridge, showing that these are prevalent defects. In some cases, the TRF1 signals from two non-homologous chromosomes were fused and appeared as a single signal (referred to as chromosome fusion; Figure 3D).

In addition to these structural defects, we also observed TRF1 foci that were not connected to the chromosomal ends (referred to as a solitary TEL) as well as chromosome ends lacking TRF1 foci (referred to as telomere erosion; Figure 3E). These phenotypes are likely to be a consequence of severe splits and bridges that lead to the separation of telomeres from their cognate chromosomal ends.

The same structural defects were observed by staining for TRF2 in spermatocytes, thus suggesting that these cytological phenotypes are not a consequence of TRF1 mislocalization but rather reflect defective telomeric dsDNA structures (Figures S3A and S3B). Further, prophase I oocytes collected from *Terb1^ΔMYB/ΔMYB^* female embryos exhibited similar telomeric defects as those observed in spermatocytes, suggesting that the function of the TERB1 MYB domain is conserved in both sexes (Figure S3C). Finally, fluorescent *in situ* hybridization using a telomeric probe confirmed the telomere length heterogeneity in *Terb1^ΔMYB/ΔMYB^* spermatocytes containing abnormally strong signals and almost no detectable signal at chromosomal ends, which represent fused and bridged telomeres and telomere erosion, respectively (Figure 3F).

### Meiotic telomere erosion does not accompany activation of the DNA damage response

The telomere defects seen in *Terb1^ΔMYB/ΔMYB^* mice are cytologically reminiscent of the canonical telomere dysfunctions reported in mitotic cells, such as the fragile telomeres caused by TRF1 depletion and the telomere deprotection and fusion caused by TRF2 depletion (Sfeir et al., 2009; Takai et al., 2003). These canonical telomere dysfunctions activate the DNA damage response (DDR) at telomeres as seen by the accumulation of DDR markers at telomeres, known as telomere-dysfunction-induced foci (TIF) (Takai et al., 2003). To determine whether *Terb1^ΔMYB/ΔMYB^* spermatocytes are TIF positive or not, we stained the spermatocytes for the presence of phosphorylated serine 139 of histone H2AX (γH2AX), one of the DDR markers. Unexpectedly, γH2AX signals only accumulated on the sex body in *Terb1^ΔMYB/ΔMYB^* spermatocytes, as well as in WT spermatocytes, but not on telomeres with structural defects or erosion (Figure 3G). These results suggest that the structural defects and erosion of telomeres seen in *Terb1^ΔMYB/ΔMYB^* mice do not accompany DDR activation and thus are qualitatively distinct phenotypes from the canonical telomere dysfunctions or deprotection that accompany TIF in mitotic cells.

### Abrogation of late-pachytene axial element remodeling

**Figure 4.**
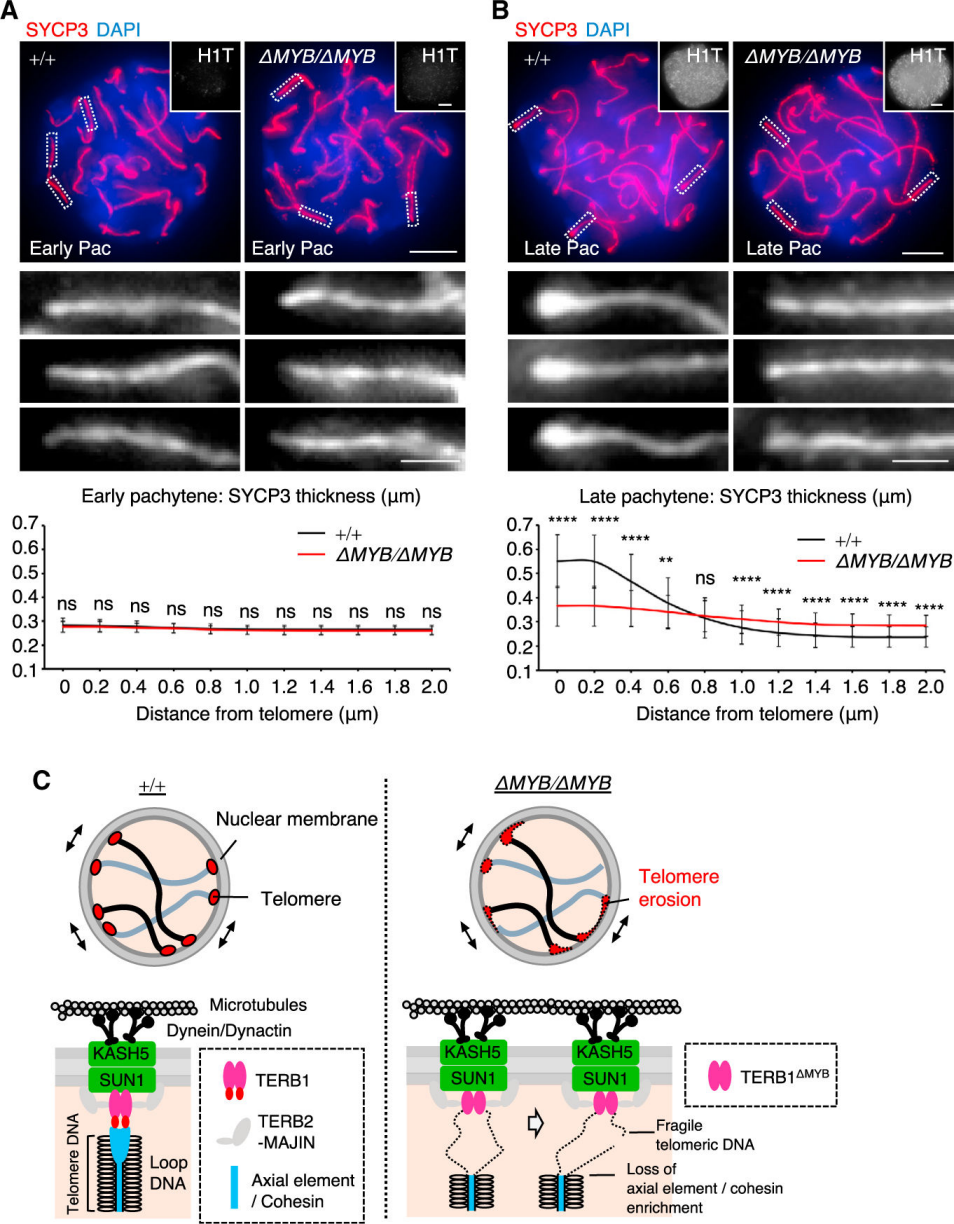
Abrogation of the late-pachytene axial element remodeling in *Terb1^ΔMYB/ΔMYB^* mice (A and B) Immunostaining of WT and *Terb1^ΔMYB/ΔMYB^* spermatocytes in early (A) and late (B) pachytene spermatocytes. The graph shows the mean thickness of SYCP3 signals at telomeres (0 μm) and at different distances on the adjacent arm region. Ten chromosomal ends from 10 cells (A) or 12 cells (B) were quantified. Error bars represent standard deviation. Scale bars, 5 μm. All analyses used two-tailed t tests. **p < 0.01; ****p < 0.0001. (C) Schematic of the telomeric defects seen in *Terb1^ΔMYB/ΔMYB^* meiocytes.

The SYCP2 and SYCP3 heterodimer is recruited onto the chromosomal axis by the cohesin axial core and forms the axial element in meiotic prophase I. In late-pachytene spermatocytes, the telomeric region of the axial element thickens, and this is conventionally used to distinguish the late-pachytene population from the early-pachytene population (Morelli et al., 2008; Zhang et al., 2019), although the underlying molecular mechanism behind this thickening has been elusive. When observing spermatocyte spreads, we noticed that the spermatocyte population with the thickened axial element ends seen in WT spermatocytes was completely absent in the testis cell suspension from *Terb1^ΔMYB/ΔMYB^* mice. To further investigate this, we stained spermatocytes with the testis-specific histone H1T, which is a specific marker of late-pachytene spermatocytes, and quantified the thickness of the SYCP3 signal at telomeres and the adjacent arm regions. WT H1T-negative early-pachytene spermatocytes showed no significant difference in the SYCP3 thickness at telomeres (0.28 μm at 0 μm from the telomeres) and the arm region (0.27 μm at 0.6 μm from the telomeres; Figure 4A). However, in WT H1T-positive late-pachytene spermatocytes, a drastic thickening of the axial element was seen at telomeres (0.55 μm at 0 μm from the telomeres) relative to the arm region (0.39 μm at 0.6 μm from the telomeres; Figure 4B). These results showed that the thickening of axial elements at telomeres indeed coincided with the early-pachytene to late-pachytene transition in WT spermatocytes. Notably, this temporal remodeling of axial elements was largely abolished in *Terb1^ΔMYB/ΔMYB^* mice, where the thickness of the axial elements at telomeres (0.36 μm at 0 μm from the telomeres) was comparable to the arm region (0.34 μm at 0.6 μm from the telomeres), even in late-pachytene spermatocytes (Figure 4B). The thickened axial element may be a structure corresponding to the conical thickening of lateral elements seen by the electron microscopic observation of the telomere-NE attachment sites (Liebe et al., 2004) and is most likely what confers structural rigidity to chromosomal ends and thus protects telomeric DNA from mechanical disruption during chromosome movements.

In line with the cytological observations, TERB1 IP from WT and *Terb1^ΔMYB/ΔMYB^* testis extracts showed that the co-immunoprecipitated SYCP3 signal was significantly reduced in the *Terb1^ΔMYB/ΔMYB^* case (Figure 3B). Together, these results suggest that the TERB1 MYB domain regulates the enrichment of cohesin and axial elements at telomeres, likely through protein-protein interactions, and this enrichment is needed to maintain telomere structures and to avoid telomere fusion and erosion (Figure 4C). Because the localization of cohesin is upstream of the recruitment of the SYCP2-SYCP3 heterodimer (Ishiguro et al., 2014), the loss of the cohesin axial core at telomeres is likely to be the primary defect that leads to the loss of axial element recruitment in *Terb1^ΔMYB/ΔMYB^* spermatocytes.

### The TERB1 MYB domain is dispensable for mouse fertility

**Figure 5.**
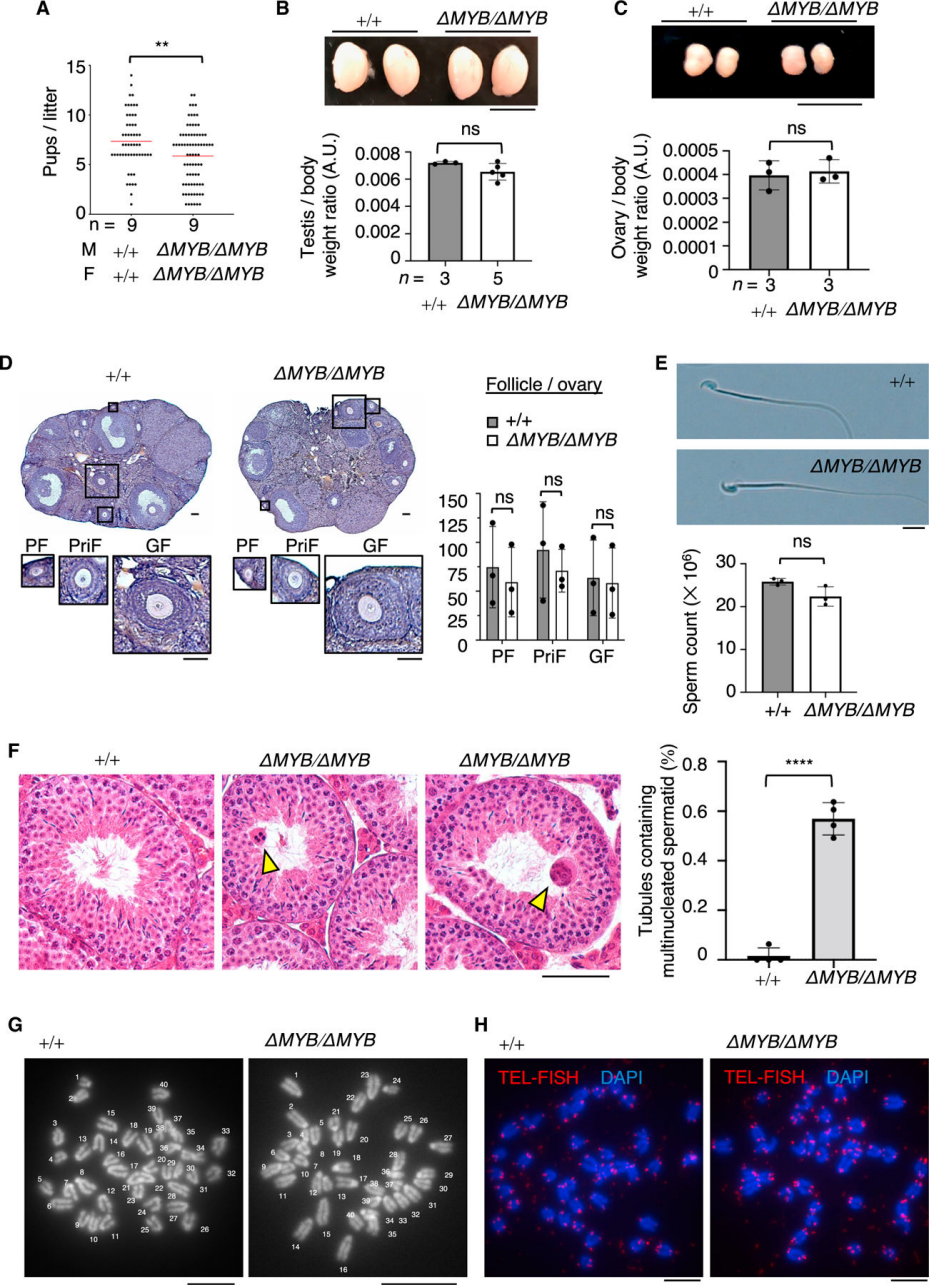
*Terb1^ΔMYB/ΔMYB^* mice are fertile (A) The average number of pups per litter. Postnatal day 60 male (M) and female (F) pairs of WT (+/+) and *Terb1^ΔMYB/ΔMYB^* mice were paired for >60 days of continuous breeding. n indicates the number of mating pairs. Two-tailed t tests; **p < 0.01. (B) Testes from WT (+/+) and *Terb1^ΔMYB/ΔMYB^* mice at 2 months of age with quantification of the testis/body weight ratio. The mean values with SD are shown. n shows the number of mice. Scale bar, 5 μm. Two-tailed t tests. (C) Ovaries from WT (+/+) and *Terb1^ΔMYB/ΔMYB^* mice at 2 months of age with quantification of the ovary/body weight ratio. The mean values with SD are shown. n shows the number of mice. Scale bar, 5 μm. Two-tailed t tests. (D) Ovary sections from PD40 WT (+/+) and *Terb1^ΔMYB/ΔMYB^* female mice stained with hematoxylin and eosin. The representative images of a primordial follicle (PF), primary follicle (PriF), and growing follicle (GF) are magnified. The graph shows the number of follicles in each ovary. The mean values of three independent experiments from three different mice with SD are shown. Scale bars, 40 μm. Two-tailed t tests. (E) The morphology of mature sperm from PD90 WT (+/+) WT and *Terb1^ΔMYB/ΔMYB^* male mice. The graph shows the number of sperm per epididymis. Mean values of three independent experiments from three different mice with SD are shown. Scale bars, 10 μm. Two-tailed t tests. (F) Testis sections from adult WT (+/+) and *Terb1^ΔMYB/ΔMYB^* mice stained with hematoxylin and eosin. Arrowheads show the multinucleated giant spermatids. Scale bar, 100 μm. The mean ratios of seminiferous tubules containing multinucleated giant spermatids were quantified from four adult WT and *Terb1^ΔMYB/ΔMYB^* mice, respectively. The mean values with SD are shown. More than 1,000 seminiferous tubules were observed for each mouse. Two-tailed t tests. ****p < 0.0001. (G) Prometaphase I chromosome spreads from WT and *Terb1^ΔMYB/ΔMYB^* mouse embryonic fibroblasts (MEFs) stained with DAPI. There were 40 chromosomes in both the WT and *Terb1^ΔMYB/ΔMYB^* MEFs, showing that these cells had a normal karyotype. Scale bars, 15 μm. (H) Prometaphase I chromosome spreads from WT and *Terb1^ΔMYB/ΔMYB^* MEFs stained with DAPI and hybridized with telomeric peptide nucleic acid (PNA) probes (TEL-FISH). Scale bars, 5 μm.

Notably, and to our surprise, the crossing of male and female *Terb1^ΔMYB/ΔMYB^* mice showed that these mice were fertile but with somewhat reduced numbers of pups per litter compared with WT pairs (Figure 5A). The sizes of adult testes and ovaries were also comparable to those of WT mice (Figures 5B and 5C). The observation of follicles and sperm in WT and *Terb1^ΔMYB/ΔMYB^* ovaries and epididymides, respectively, confirmed that there was no significant difference in their numbers or morphologies between WT and *Terb1^ΔMYB/ΔMYB^* mice (Figures 5D and 5E). These results are in stark contrast to the phenotypes seen in *Terb1*-null mice, where both male and female mice suffer from complete infertility with degenerated reproductive organs (Shibuya et al., 2014).

While the MYB domain of TERB1 is dispensable for fertility, inspection of *Terb1^ΔMYB/ΔMYB^* testis sections showed the appearance of multinucleated spermatids within the seminiferous tubules, which were rarely observed in WT mice (Figure 5F). Because the formation of dicentric chromosomes after telomere fusion is a prevalent cause of chromosomal bridges in anaphase (Barra and Fachinetti, 2018), the observed multinucleated spermatids in *Terb1^ΔMYB/ΔMYB^* testes are likely to be a consequence of the telomere fusion and subsequent chromosomal nondisjunction. However, the proportion of seminiferous tubules with multinucleated spermatids was only 0.6% in *Terb1^ΔMYB/ΔMYB^* testis, and this combined with the subfertility phenotype made it clear that most of the germ cells with the telomere defects still matured into functional germ cells.

Further, the analysis of embryonic fibroblasts derived from WT and *Terb1^ΔMYB/ΔMYB^* mice showed the presence of normal chromosome numbers and telomere structures without any end-to-end chromosome fusion (Figures 5G and 5H). These results suggest that the prevalent telomere bridges and fusions seen in meiotic prophase I in *Terb1^ΔMYB/ΔMYB^* mice are largely resolved into individual chromosomes during meiotic cell division and do not affect the karyotype or the telomeric structure in the next generation, at least under normal laboratory conditions.

## DISCUSSION

The MYB domain was initially characterized as a sequence-specific DNA-binding domain in the MYB transcription factors and later was found in a number of telomeric dsDNA-binding proteins in a wide variety of metazoan species (Bilaud et al., 1996; Oh and Reddy, 1999). Even though the MYB domains generally function in dsDNA binding, they have sometimes acquired protein binding roles during evolution (Grotewold et al., 2000; Ko et al., 2008). TERB1 is a meiosis-specific telomere-binding protein that functions in telomere-driven chromosome movements in meiotic prophase I (Shibuya et al., 2014). TERB1 has a MYB domain on its C terminus, which is widely conserved in metazoan species, but its role has been elusive (da Cruz et al., 2020). Our structural modeling showed that the MYB domain of TERB1 shares overall homology with that of canonical telomeric factors TRF1/2; however, the key DNA recognition sites found in TRF1/2 are different in the TERB1 MYB domain. Consistent with this, the TERB1 MYB domain has lost its DNA-binding activity.

The analysis of *Terb1^ΔMYB/ΔMYB^* mice demonstrated that the TERB1 MYB domain is dispensable for the localization of TERB1 and the downstream TERB2-MAJIN complex at meiotic telomeres. This is in contrast to the situation of canonical telomeric factors, such as TRF1 and TRF2, i.e., their MYB domains directly bind to telomeric dsDNA and are indispensable for the telomeric localization of TRF1/2 and the downstream shelterin components (Palm and de Lange, 2008).

Consistent with the normal localization of the TERB1-TERB2-MAJIN ternary complex, the *Terb1^ΔMYB/ΔMYB^* mice achieved normal telomere attachment to the NE, homolog synapsis, and recombination. Instead, we found that the TERB1 MYB domain is required for the interaction with cohesin and axial element proteins and that the loss of cohesin and axial elements from telomeres in *Terb1^ΔMYB/ΔMYB^* mice resulted in aberrant telomere structures, such as splits and bridges, that culminated in the separation of the telomeric DNA from the cognate chromosomal ends. Hence, we concluded that the TERB1 MYB domain has specific roles in the protection of telomeric DNA from aberrant erosion likely during chromosome movements. Of note, the TERB1 MYB domain also ensures the remodeling of the axial element at chromosomal ends in the early-to-late pachytene transition. During this transition, the shelterin complex relocates from the very ends of chromosomes to the surrounding ring-shaped structures in a manner dependent on the activation of cyclin-dependent kinases, a phenomenon referred to as the cap exchange, as shelterin is apparently replaced by the TERB1-TERB2-MAJIN complex at chromosomal ends (Shibuya et al., 2015). Considering that the axial element remodeling coincides with the cap exchange, such remodeling is probably associated with the formation of a unique chromatin environment that facilitates end protection (e.g., by forming a rigid chromatin environment to protect shelterin-free telomeric DNA from aberrant erosion or recombination). The physiological function of the cap exchange remains enigmatic, and further studies are needed to reveal the molecular basis of this intriguing cell biological observation.

The aberrant telomere structures were also seen in *Terb1* knockout (KO) mice, while *Terb1* KO mice showed additional meiotic defects, including defects in telomere attachment to the NE, homologous synapsis, and recombination, which culminate in complete meiosis arrest at the zygotene stage (Shibuya et al., 2014). Therefore, the *Terb1^ΔMYB/ΔMYB^* point mutation is a separation-of-function mutation that rescues the major meiotic abnormalities in the *Terb1* KO mice, except for the telomere structural defects. Similar telomere structural defects were also reported in mice lacking the meiosis-specific cohesin subunit SMC1β, and these mice showed mislocalization of meiotic cohesin specifically from telomeres similar to the *Terb1* KO mice and the *Terb1^ΔMYB/ΔMYB^* mice (Adelfalk et al., 2009).

How the TERB1 MYB domain promotes the localization of cohesin at telomeres is still unknown. Our previous study using the yeast two-hybrid system showed that the TERB1 C terminus, which includes the MYB domain, directly binds to SA3, a meiosis-specific cohesin component (Shibuya et al., 2014), suggesting that the direct protein-protein interaction between SA3 and the TERB1 MYB domain might be required for the targeting of cohesin to meiotic telomeres. Of note, the mitotic telomeric factor TRF1 directly binds to SA1, a mitotic counterpart of SA3, and targets SA1 to mitotic telomeres in order to maintain telomeric cohesion (Lin et al., 2016). These analogous findings suggest that the targeting of cohesin to telomeres through direct protein interactions is a conserved role of the MYB-containing telomeric factors.

It is surprising that the *Terb1^ΔMYB/ΔMYB^* mice are subfertile and can have healthy offspring without any detectable telomeric abnormalities or chromosomal instability in their somatic cells. These observations suggest that the telomeric defects seen in the meiocytes are somehow repaired in the later step of spermiogenesis or in early embryogenesis, implying the presence of some active telomere repair pathway after meiosis. One candidate pathway is the activation of telomerase-dependent telomere lengthening. While telomerase activity in humans is restricted to certain cell types, such as stem cells, germ cells, or cancer cells, and is not detectable in most somatic cells, laboratory mice have ubiquitous telomerase activity even in their somatic cells and thus have extremely long telomeres compared with other vertebrate species (up to 10 times longer than humans; Gomes et al., 2011). We speculate that these long telomeres and high telomerase activity might be sufficient to restore meiotic telomere erosion during early development, resulting in no apparent transgenerational defects in *Terb1^ΔMYB/ΔMYB^* mice. Nonetheless, considering the perfect conservation of the TERB1 MYB domain across a broad spectrum of metazoan phyla, the TERB1 MYB domain is likely to be important for the maintenance of telomeric DNA and thus for genomic integrity on longer evolutionary timescales, especially in non-rodent species with limited telomerase activity. It will be interesting in future experiments to investigate the impact of TERB1 MYB domain deletion in other model organisms and mice with compromised telomerase activity.

## Limitations of the study

We propose that cohesin and axial elements enriched at meiotic telomeres by the TERB1 MYB domain confer structural rigidity to chromosomal ends, which likely protects telomeric DNA from mechanical disruption by the chromosome movement forces. This hypothesis could be further addressed by artificially stopping the chromosome movements in the *Terb1^ΔMYB/ΔMYB^* mice, which could rescue the structural defects. However, the introduction of movement-defective mutations, such as *Sun1*- or *Kash5*-null alleles, will cause severe meiotic defects, including defects in telomere attachment to the NE and zygotene-stage arrest (Ding et al., 2007; Horn et al., 2013), which will hinder the phenotypic comparison with the *Terb1^ΔMYB/ΔMYB^* mice. Alternatively, the live-cell observation of meiotic telomeres in the *Terb1^ΔMYB/ΔMYB^* mice could be useful for detecting the telomere disruption coupled with its movements along the NE.

The reduction of SMC3 localization at telomeres in the *Terb1^ΔMYB/ΔMYB^* mice could be in part explained by the reduction of TERB1-TERB2-MAJIN itself from telomeres rather than the MYB deletion-specific effect. However, our quantification showed that the signal intensity of TERB1^ΔMYB^ was around 66% of WT levels in the *Terb1^ΔMYB/ΔMYB^* mice (Figure 2A), while the reduction of SMC3 was much more drastic (44% of the WT levels; Figure 3A). Furthermore, our previous study showed that even overexpression of TERB1^ΔMYB^ protein by the strong exogenous CAG promoter failed to rescue SMC3 mislocalization from telomeres in *Terb1* KO spermatocytes (Zhang et al., 2017). These results support our conclusion that the TERB1 MYB domain is responsible for the telomere localization of SMC3.

The *Terb1^ΔMYB/ΔMYB^* mice have been maintained for more than 10 generations in our laboratory conditions without any obvious somatic defects. Analyses of the *Terb1^ΔMYB/ΔMYB^* mice over a much longer timescale or the *Terb1^ΔMYB/ΔMYB^* mutant models in non-rodent animal models with shorter telomeres might provide further evidence for the role of the TERB1 MYB domain in the transgenerational maintenance of telomeric DNA and genomic integrity.

## STAR*METHODS

### RESOURCE AVAILABILITY

#### Lead contact

Further information and requests for resources and reagents should be directed to and will be fulfilled by the lead contact, Hiroki Shibuya (hiroki.shibuya@gu.se).

#### Materials availability

Plasmids and mouse samples generated in this study are available from the lead contact upon request.

#### Data and code availability

- The data reported in this paper will be shared by the lead contact upon request.
- This paper does not report original code.
- Any additional information required to reanalyze the data reported in this work paper is available from the lead contact upon request.

### EXPERIMENTAL MODEL AND SUBJECT DETAILS

Mice were congenic with the C57BL/6J background. Mature adult mice in age from 2 – 6 moths were used. All animal experiments were approved by the Institutional Animal Care and Use Committee (#1316/18). For the generation of sgRNA, the double-stranded oligonucleotide (5’-TCCCAGTTCCAGGCCAGAG-3’) was inserted into to a pUC57-sgRNA expression vector, and *in vitro* transcription was performed with a MEGAshortscript kit (Ambion; AM1354). For the identification of founder mice, the extracted DNA was amplified with the following primers and the PCR products were sequenced: Forward; 5’-CACTGATTCCACAGGTTGTTTC-3’, Reverse; 5’-CACAGAGAAGAATACCAACATTTGT-3’.

### METHOD DETAILS

#### Histological analysis

Testes were fixed in Bouin’s fixative for 24 h at room temperature and embedded into paraffin blocks. Slices of 8 μm thickness were stained with hematoxylin and eosin. TUNEL analysis was carried out with an ApopTag Plus In Situ Apoptosis Fluorescein Detection Kit (S 7111; Millipore).

#### Follicle counting

Ovaries from postnatal day (PD) 40 female mice were fixed in 4% paraformaldehyde, dehydrated, and embedded in paraffin. Paraffin-embedded ovaries were then cut into 8-mm serial sections and stained with hematoxylin and eosin. The follicles were classified into three stages (primordial, primary, and growing follicles) and counted from the middle continuous sections.

#### Sperm counting

PD90 male mice were euthanized, their epididymides were isolated, and, after making multiple incisions, sperm were released into 1 ml Milli-Q water for 30 min at 37°C in a 5% CO_2_ incubator. A hemocytometer was used for sperm counting.

#### Antibodies

The following antibodies were used: rabbit antibodies against TERB1 (Shibuya et al., 2014), TERB2 (Shibuya et al., 2015), MAJIN (Shibuya et al., 2015), SYCE3 (Zhang et al., 2019), TRF2 (Novus Biologicals; NB110–57130), γH2AX (Abcam; ab11174), and SMC3 (Abcam; ab9263); mouse antibodies against TRF1 (Shibuya et al., 2014), β-actin (Sigma; A2228–100UL), and MLH1 (BD Biosciences; 51–1327GR); rat antibody against RPA2 (Cell Signaling Technology; 2208); guinea pig antiserum against histone H1T (Inselman et al., 2003); goat antibody against Lamin B (Santa Cruz Biotechnology; sc-6216); and chicken antibody against SYCP3 (Zhang et al., 2019).

#### Immunostaining of spermatocytes

Testis cell suspensions were prepared and washed in PBS, centrifuged, and resuspended in hypotonic buffer (30 mM Tris (pH 7.5), 17 mM trisodium citrate, 5 mM EDTA, and 50 mM sucrose) followed by centrifugation and resuspension in 100 mM sucrose. The cell suspensions were placed on slides in the same volume of fixation buffer (1% paraformaldehyde and 0.1% Triton X-100), fixed for 3 h at room temperature, and air dried. For immunostaining, the slides were incubated with primary antibodies in PBS containing 5% BSA for 2 h and then with Alexa Fluor 488-, 594-, or 647-conjugated secondary antibodies (1:1,000 dilution, Invitrogen) for 1 h at room temperature. The slides were washed with PBS and mounted with VECTASHIELD medium with DAPI (Vector Laboratories). The Z-stack images were acquired with a 0.45 mm step size. The signal quantifications were performed using the original 3D images for SMC3 or the projections of Z-stack images for the rest. Signal intensities were measured using the SoftWorx Data Inspector tool. Telomeric regions were defined as the end of the SYCP3 signals, and the signals were quantified. The neighboring background signals were measured for each data point and subtracted.

#### Preparation of testis extract and IP

Testes were suspended in extraction buffer (20 mM Tris-HCl (pH 7.5), 50 mM KCl, 0.4 mM EDTA, 5 mM MgCl_2_, 10% glycerol, 0.1% Triton X-100, and 1 mM β-mercaptoethanol) supplemented with cOmplete Protease Inhibitor (Roche) and Phosphatase Inhibitor (Roche). After homogenization, the cell extract was centrifuged and the pellet was lysed with high-salt buffer (20 mM HEPES (pH 7.0), 400 mM KCl, 5 mM MgCl_2_, 10% glycerol, 0.1% Triton X-100, and 1 mM β-mercaptoethanol) supplemented with cOmplete Protease Inhibitor (Roche) and Phosphatase Inhibitor (Roche). After centrifugation, the supernatant was collected, supplemented with Dynabeads protein A (Thermo Fisher Scientific), conjugated with 80 μg of anti-TERB1 antibody or control IgG as the negative control, and incubated for 6 h at 4°C. The beads were washed with high-salt buffer. The samples were eluted with 0.1 M glycine (pH 2.5).

#### Protein purification

The His-Smt3-hTRF2^myb^ pET28B vector was used to express amino acid 446–500 of human TRF2 as a 10× His-tagged and Smt3-tagged fusion protein in BL21 (DE3) *E. coli* cells after induction with 0.1 mM isopropyl β-D-1-thiogalactopyranoside (IPTG) at 25°C for 14–18 h. To lyse, cell pellets were first resuspended in lysis buffer (25 mM Tris-HCl (pH 8), 500 mM NaCl, 10 mM 2-mercaptoethanol, 0.1 mM EDTA, 1 mM PMSF, and 1× protease inhibitor (cOmplete Protease Inhibitor Cocktail, Roche) and then sonicated. The lysate was clarified via centrifugation, and the clarified supernatant was incubated with nickel-NTA agarose beads (Qiagen) at 4°C for 2 h. Beads were collected by gravity flow and washed five times with wash buffer (25 mM Tri-HCl (pH 8), 150 mM NaCl, 10 mM 2-mercaptoethanol), and 0.5 mg Ulp1 protease was added to the washed beads and incubated overnight at 4°C to cleave off the His-Smt3 tag. Flowthrough fractions from the column post-cleavage were pooled and subjected to Superdex 75 size-exclusion chromatography to obtain essentially homogenous hTRF2^myb^ for use in the EMSA analysis. The wild type and mutant TERB1 MYB domains were purified similarly using His-Smt3-hTERB1^myb^ pET28B plasmids. The mTERB1 MYB protein was purified as a 6× His-tagged fusion protein using nickel-NTA affinity chromatography and was assayed as such without cleavage of the polyhistidine tag.

### EMSA

The DNA probes containing eight telomere repeats (TTAGGG) or scrambled sequences were radio-labeled with [γ-^32^P] ATP by T4 polynucleotide kinase (New England Biolabs). Binding reactions included 150 nM of probe DNA and varying concentration of purified proteins in 10 μl of binding buffer (50 mM Tris-HCl (pH 8.0), 6.5% glycerol, 20 mM NaCl, and 5 mM DTT). Mixtures were incubated for 30 min on ice and electrophoresed through a 4–20% Novex™ TBE non-denaturing gel (Life Technologies) at 200 V for 1 h.

#### Structure modeling

The homology model for human TERB1 MYB was generated in Swiss-Model (Guex et al., 2009). The model was built using the highly similar mouse TERB1 MYB structure (PDB ID: 1X58) as the template.

#### Cell culture

Mouse embryonic fibroblasts were isolated from embryonic day 13.5 embryos and cultured in DME containing 10% bovine calf serum at 37°C and 5% CO_2_. For synchronization, cells were treated for 14 h with 0.5 μg/mL nocodazole.

#### Fluorescent *in situ* hybridization assay

Spermatocyte or mouse embryonic fibroblast spreads were treated with RNase A (100 mg/mL) at 37°C for 30 min in 2× saline sodium citrate (SSC), denatured at 85°C for 10 min with Cy3-labeled TelC (CCCTAA)_3_ PNA probe (Panagene), and hybridized for 4 h at 37°C. Preparations were washed twice in 50% formamide/0.5× SSC and then washed twice in 1× SSC at 42°C for 5 min each time.

### QUANTIFICATION AND STATISTICAL ANALYSIS

Statistical analysis was performed using GraphPad Prism or Microsoft EXCEL. No statistical method was used to determine sample size, and sample sizes are consistent with those reported in similar studies. For comparisons of two groups, two-tailed Student’s t test was used (Figures 2A–2C, 2F, 3A, 3C–3E, 4A, 4B, 5A–5F, S2A, S2D, S3A–S3C). For comparison of distribution, Mann-Whitney test was used (Figure 3F). For multiple test subjects, one-way ANOVA followed by Dunnett’s test was used (Figure 2E). The statistical details of experiments can be found in the figure legends and figures.

## Supplementary Material

1

## Figures and Tables

**KEY RESOURCES TABLE:** 

REAGENT or RESOURCE	SOURCE	IDENTIFIER

Antibodies		

Rabbit Anti-TERB1	Hiroki Shibuya lab	N/A
Rabbit Anti-TERB2	Hiroki Shibuya lab	N/A
Rabbit Anti-MAJIN	Hiroki Shibuya lab	N/A
Rabbit Anti-SYCE3	Hiroki Shibuya lab	N/A
Rabbit Anti-TRF2	Novus Biologicals	NB110-57130, M-1
Rabbit Anti-SMC3	Abcam	ab9263, GR3221084-4
Rabbit Anti-γH2AX	Abcam	Ab11174, GR2948890-8
Mouse Anti-TRF1	Hiroki Shibuya lab	N/A
Mouse Anti-β-actin	Sigma	A2228-200UL, 067M4856V
Mouse Anti-MLH1	BD Biosciences	51-1327GR, 4136717
Rat Anti-RPA2	Cell Signaling Technology	2208S, 3
Guinea pig Anti-histone H1T	Mary Ann Handel lab	N/A
Goat Anti-Lamin B	Santa Cruz Biotechnology	sc-6216, F1715
Chicken Anti-SYCP3	Hiroki Shibuya lab	N/A
Donkey Anti-Rabbit Alexa 488	Invitrogen	Cat#A21206
Donkey Anti-Rabbit Alexa 594	Invitrogen	Cat#A21207
Donkey Anti-Mouse Alexa 594	Invitrogen	Cat#A21203
Donkey Anti-Mouse Alexa 488	Invitrogen	Cat#A21202
Goat Anti-Chicken Alexa 647	Invitrogen	Cat#A21449
Goat Anti-Chicken Alexa 594	Invitrogen	Cat#A11042
Donkey Anti-Rat Alexa 594	Invitrogen	Cat#A21209
Donkey Anti-Goat Alexa 488	Invitrogen	Cat#A32814
Peroxidase Goat Anti-Mouse IgG	Bio Rad	Cat#170-6516
Peroxidase Goat Anti-Rabbit IgG	Bio Rad	Cat#170-6515

Bacterial and virus strains		

Subcloning efficiency™ DH5α™ competent cells	Thermo Fisher Scientific	Cat#18265017
One shot™ BL21(DE3)pLysS chemically competent *E. coli*	Thermo Fisher Scientific	Cat#C606003

Chemicals, peptides, and recombinant proteins		

Bouin’s solution	Sigma	Cat#HT10132-1L
Vectashield	Vector	Cat#H-1200
Isopropyl beta-D-thio galactopyranoside (IPTG)	Goldbio	Cat#I2481
Protease Inhibitor Cocktail Tablets	Roche	Cat#4693132001
Imidazole	Fischer scientific	Cat#AC301872500
DTT [Dithiothreitol]	Melford	Cat#D11000
Amylose Resin	New England Biolabs	Cat#E8021S

Critical commercial assays		

ApopTag Plus In Situ Apoptosis Fluorescein Detection Kit	Millipore	Cat#S7111

Experimental models: Cell lines		

C57BL/6J mouse embryonic fibroblast primary culture	Hiroki Shibuya lab	N/A
C57BL/6J *Terb1^ΔMYB/ΔMYB^* mouse embryonic fibroblast primary culture	Hiroki Shibuya lab	N/A

Experimental models: Organisms/strains		

Mouse: C57BL/6J	Jackson Lab	N/A
Mouse: C57BL/6J *Terb1^−/−^*	Hiroki Shibuya lab	N/A
Mouse: C57BL/6J *Terb1^ΔMYB/ΔMYB^*	Hiroki Shibuya lab	N/A

Oligonucleotides		

sgRNA oligo: 5’-TCCCAGTTCCAGGCCAGAG-3’	This study	N/A
*Terb1^ΔMYB/ΔMYB^* Genotype For: 5’-CACTGATTCCACAGGTTGTTTC-3’	This study	N/A
*Terb1^ΔMYB/ΔMYB^* Genotype Rev: 5’- CACAGAGAAGAATACCAACATTTGT-3’	This study	N/A
dsDNA_telo_8-mer (DNA duplex) Forward strand sequence: 5’-TTAGGGTTAGGGTTAGGGTTAGGGTTAGGGTTAGGGTTAGGGTTAGGG-3’	This study	N/A
dsDNA _scrambled_8-mer (DNA duplex) Forward strand sequence: 5’-GACAGCGATGAGAACTAATTCGTGTGCTTGCTGACTGATATCGTGACT-3’	This study	N/A

Recombinant DNA		

pET28B-His-Smt3-hTRF2^myb^	This study	N/A
pET28B-His-Smt3-hTERB1^myb^	This study	N/A
pET28C-His-mTERB1^myb^	This study	N/A
pET28B-His-Smt3-hTERB1^myb^(A712K)	This study	N/A
pET28B-His-Smt3-hTERB1^myb^(H713D)	This study	N/A
pET28B-His-Smt3-hTERB1^myb^(H716R)	This study	N/A

Software and algorithms		

Excel	Microsoft	https://products.office.com/
GraphPad Prism 7	GraphPad Software	https://www.graphpad.com/scientific-software/prism/
SoftWoRx	GE Healthcare Life Science	http://www.gelifesciences.com/webapp/wcs/stores/servlet/productById/en/GELifeSciences-se/29065728

## Highlights

- The TERB1 MYB domain has lost its canonical DNA-binding activity
- The TERB1 MYB domain localizes cohesin and axial element proteins at telomeres
- The TERB1 MYB domain remodels axial elements in the early-to-late pachytene transition
- The TERB1 MYB domain suppresses meiotic telomere erosion
