# Supplementary material for: The TERB1 MYB domain suppresses telomere erosion in meiotic prophase I
Source: Cell Rep. Author manuscript; Available in PMC 2022 Feb 24. (PMC8867601; doi:10.1016/j.celrep.2021.110289)
Supplement: 1 [file NIHMS1774732-supplement-1.pdf]

Cell Reports, Volume *38*

Supplemental information

The TERB1 MYB domain suppresses telomere erosion in meiotic prophase I

Kexin Zhang, Agata Tarczykowska, Deepesh Kumar Gupta, Devon F. Pendlebury, Cassandra Zuckerman, Jayakrishnan Nandakumar, and Hiroki Shibuya

Supplementary information

The TERB1 MYB domain suppresses telomere erosion in meiotic prophase I

Zhang. K, et al.

Figure S1

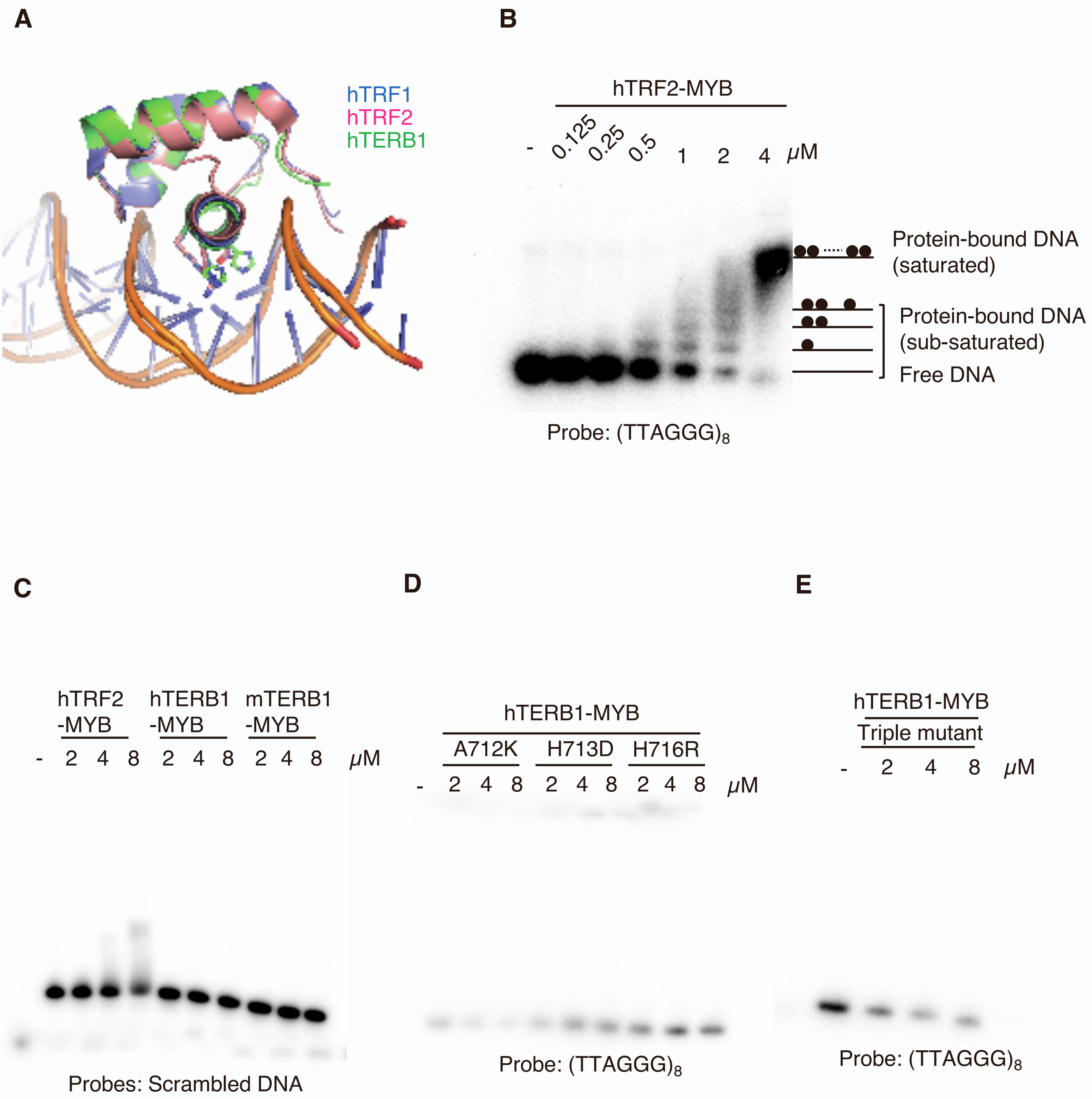

Figure S1. The TERB1 MYB domain is not a DNA-binding domain
(**A**) Overlay of the crystal structures of dsDNA-bound human TRF1-MYB (PDB ID: 1W0T), human TRF2-MYB (PDB ID: 1W0U), and a homology model of human TERB1-MYB. The homology model of the human TERB1 MYB domain was obtained from the structure of the mouse TERB1 MYB domain (PDB ID: 1X58). (**B**) EMSA assay with isolated MYB domain from hTRF2 using telomeric DNA probe. (**C**) EMSA assay with isolated MYB domains from hTRF2, hTERB1, and mTERB1 using the scrambled DNA sequence (5' – GACAGCGAT GAGAACTAATTCGTGTGCTTGCTGACTGATATCGTGACT – 3'). (**D** and **E**) EMSA assay with isolated MYB domains from hTERB1with three single substitutions (**D**) and a triple mutant (**E**) using telomeric DNA probe.

Figure S2

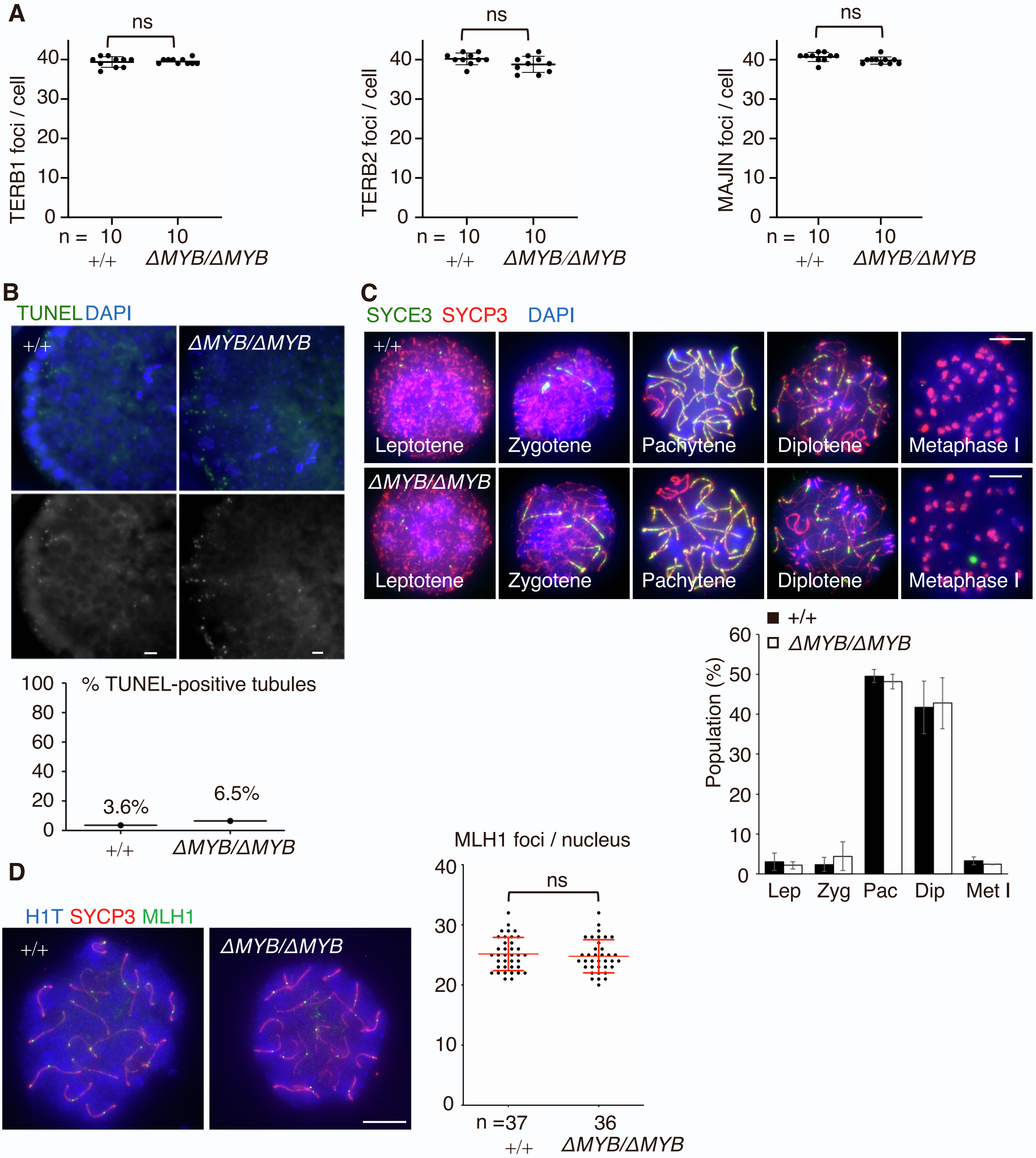

Figure S2. Homologous synapsis and recombination are intact in *Terb1*$^{ΔMYB/ΔMYB}$ spermatocytes
(**A**). The number of TERB1, TERB2, and MAJIN foci per pachytene cell (n = 10 cells). The mean values with SD are shown. (**B**). Testis sections from 8-week-old WT and *Terb1*$^{ΔMYB/ΔMYB}$ testes stained with TUNEL and DAPI. The percentages of TUNEL-positive seminiferous tubules (those containing more than three TUNEL-positive cells) were quantified. n shows the analyzed seminiferous tubule number. (**C**). Immunostaining of WT and *Terb1*$^{ΔMYB/ΔMYB}$ spermatocytes. To quantify the populations of each meiotic prophase substage in testis cell suspensions, SYCP3-positive spermatocytes (992 cells for WT and 1,016 cells for *Terb1*$^{ΔMYB/ΔMYB}$, pooled from two mice for each genotype) were classified into the following substages: Lep, leptotene (no SYCE3); Zyg, zygotene (partially assembled SYCE); Pac, pachytene (fully assembled SYCE3); Dip, diplotene (disassembled SYCE3); and Met I, metaphase I (SYCP3 accumulations at centromeres but no SYCE3). The mean values of two independent experiments from two different mice are shown. Error bars show the SD. (**D**). Immunostaining of WT and *Terb1*$^{ΔMYB/ΔMYB}$ late-pachytene spermatocytes. The graph shows the number of MLH1 foci associated with the chromosome axes. The mean values with SD are shown. n shows the analyzed cell number pooled from two mice for each genotype. All analyses used two-tailed t tests. ns, not significant. Scale bar, 5 µm.

Figure S3

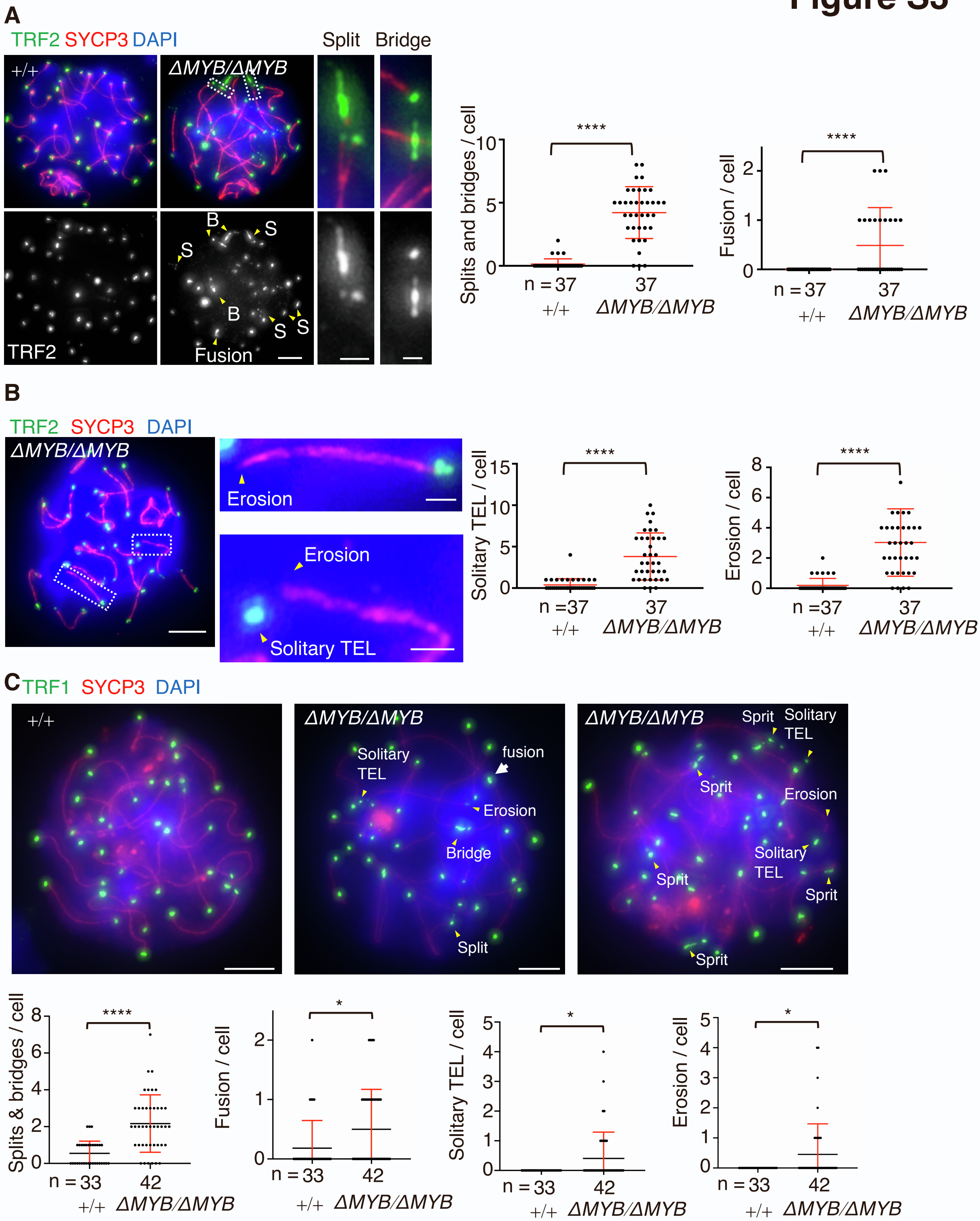

Figure S3. Telomere structural defects in $Terb1^{\Delta MYB/\Delta MYB}$ spermatocytes
(**A-B**). Immunostaining of WT and $Terb1^{\Delta MYB/\Delta MYB}$ spermatocytes. The numbers of splits (S) and bridges (B) were quantified in (**A**). The numbers of erosion and solitary telomere (TEL) were quantified in (**B**). The mean numbers with SDs were shown. n shows the analyzed cell number pooled from two mice for each genotype. (**C**) Immunostaining of WT and $Terb1^{\Delta MYB/\Delta MYB}$ oocytes collected from embryonic day 19 ovaries. The numbers of splits, bridges, and fusion, solitary, and erosion telomeres were quantified. The mean numbers with SD per cell are shown. n shows the analyzed cell number pooled from more than 5 embryos for each genotype. All analyses used two-tailed t-tests. *$p < 0.05$, ****$p < 0.0001$. Scale bar, 5 μm (1 μm in the magnified panel).
